# Evaluating IL-21 as a Potential Therapeutic Target in Crohn's Disease

**DOI:** 10.1155/2018/5962624

**Published:** 2018-04-10

**Authors:** Thomas Lindebo Holm, Ditte Tornehave, Henrik Søndergaard, Peter Helding Kvist, Bodil-Cecilie Sondergaard, Lene Hansen, Mette Brunsgaard Hermit, Kristine Holgersen, Sandra Vergo, Klaus Stensgaard Frederiksen, Claus Haase, Dorthe Lundsgaard

**Affiliations:** ^1^Global Research, Novo Nordisk A/S, Maaloev, Denmark; ^2^Novo Nordisk LIFE In Vivo Pharmacology Centre, Frederiksberg, Denmark

## Abstract

**Background and Aim:**

Interleukin-21 (IL-21) is primarily a T cell-derived cytokine; it is upregulated in patients with Crohn's Disease (CD) and could be a potential new therapeutic target in CD.

**Methods:**

In human material, IL-21 and IL-21R expression was investigated by in situ hybridization (ISH) and immunohistochemistry (IHC) in noninflammatory bowel disease (non-IBD) controls and patients with CD. The pathologic role of IL-21 was examined in murine models of T cell-dependent and T cell-independent colitis, either with a neutralizing monoclonal antibody against IL-21 or with the transfer of CD4^+^CD45RB^high^IL-21R^−/−^ T cells. Colonic pathology was examined by endoscopy, histopathology, IHC, ELISA, and Luminex.

**Results:**

In the human intestine, IL-21 and IL-21R mRNA and protein-expressing cells were observed in the mucosa, in lymphoid aggregates of submucosa in non-IBD controls, and in lymphoid aggregates of muscularis externa in patients with CD. IL-21 expression was most abundant in germinal centers (GCs) of the lymphoid aggregates, and IL-21R expression assessed semiquantitatively, was significantly higher in patients with CD compared to non-IBD controls. Following prophylactic and interventive anti-IL-21 mAb treatment in the adoptive transfer (AdTr) model, clinical and pathological parameters were significantly reduced. The most persistent finding was a reduction in colonic infiltrating neutrophils. As well, Rag2^−/−^ mice receiving CD4^+^CD45RB^high^IL-21R^−/−^ T cells developed less severe colitis compared to Rag2^−/−^ mice receiving CD4^+^CD45RB^high^IL-21R^+/+^ T cells. No effect of reduced IL-21 signalling was observed in T cell-independent colitis.

**Conclusion:**

Our study shows that patients with CD have significant expression of IL-21 and IL-21R in the gut. As well, we show that neutralization of IL-21 in experimental T cell-driven colitis is associated with a reduction in clinical and pathological findings. This amelioration seems to be associated with a reduction in colon-infiltrating neutrophils.

## 1. Introduction

Inflammatory bowel disease (IBD) is a refractory chronic inflammatory disease in the intestine. The disease is divided into ulcerative colitis (UC) and Crohn's disease (CD). IBD is defined by clinical symptoms and radiological, endoscopic, and histopathological findings [1, 2]. Although its pathogenesis is still unclear, many studies have suggested it is a multifactorial disease involving genetic and environmental factors that cause an abnormal immune response to the gut microflora [3]. Differences in the histopathology of CD and UC clearly exist. Where UC is characterized by diffuse inflammation confined to the mucosa of the colon and rectum [2], the inflammation of CD is discontinuous and transmural and can affect the entire gastrointestinal tract [1]. Patients with CD often present complications like intestinal strictures, fistulas, and abscesses which make disease management challenging [1]. Within the last 15 years, tumour necrosis factor *α* (TNF*α*) antagonists have transformed the medical treatment of moderate to severe CD, even though a significant proportion of patients with CD do not respond adequately to treatment with these agents. Primary and secondary nonresponders to anti-TNF*α* therapy present a clinical challenge and require dose adjustment or switch to another medication. The efficacy of the treatment of anti-TNF*α*-resistant CD appears to be quite modest [4]. Thus, new drug candidates are clearly needed for the anti-TNF*α* nonresponders.

Disruption of the CD4^+^ T cell balance is an important part of CD pathogenesis [5, 6], and cytokines are principal mediators orchestrating this disturbance [5]. Thus, various cytokines have been targeted or suggested as targets for the treatment of CD [7].

IL-21 is predominantly expressed by activated Th17 cells [8, 9], T follicular helper cells [10] and NKT cells [11, 12]. IL-21 signals through a receptor composed of a specific subunit, termed IL-21R, and the common *γ*-chain subunit, shared with IL-2, IL-4, IL-7, IL-9, and IL-15 receptors [13, 14]. IL-21R is highly expressed on T and B lymphocytes [15, 16], natural killer (NK) cells, and some nonhematopoietic cells, all of which functionally respond to IL-21 [13, 17]. Previous studies have shown that IL-21 is important in the regulation of immunoglobulin synthesis by plasma cells [18], cytotoxic activity of NK cells, and CD8^+^ T cells [17]. As well, IL-21 has been reported to be a key modulator of TGF-*β* signalling, leading to reciprocal differentiation of Treg and Th17 cells [19, 20]. During T cell differentiation, IL-21 is believed to sustain IL-23R expression, which then allows increased cellular response to IL-23 [11]. IL-21 may also act through the induction of the transcription factor retinoic acid receptor-related orphan receptor (ROR*γ*t) [8, 21]. In IBD patients, IL-21 has been reported to be expressed by IFN-*γ*-producing CD4^+^ T cells [22], as well as Th17 cells [6, 23]. In the gut, Th17 cells can have tissue-protective effects and enhance epithelial barrier function, as well as increasing the synthesis of extracellular matrix-degrading proteases, inflammatory cytokines, chemokines, and adhesion molecules and recruitment of neutrophils [21, 24]. In patients with CD, some of the IL-17A-producing cells coexpress IFN-*γ* and/or FoxP3 and may undergo transformation into typical Th1 or Treg cells dependent on the costimulatory environment [25, 26].

In IBD, it is currently unknown whether IL-21 directly affects Treg proliferation, differentiation, and suppression or reduces the frequency of FoxP3-positive cells by promoting Th17 cells.

Enhanced expression of IL-21 and/or IL-21R has been documented in various diseases including CD, UC, and celiac disease [11, 22, 24, 27, 28]. The upregulation of T cell-secreted IL-21 has in particular been described in patients with CD and celiac disease [22, 27]. Traditionally, CD can be categorized into ileum, ileocolonic, and colon subtypes, based on disease location [1]. Still, these subtypes behave similarly in terms of IL-21 upregulation [29], indicating that upregulation of IL-21 may be a general mechanism in CD. Moreover, there is evidence that the blockade of IL-21 signalling by either gene-targeting studies or IL-21R-Fc treatment limits the severity of inflammation in experimental models of immune-mediated diseases [11, 30–34]. In these studies, IL-21 has been described to work through a modulation of immune cell trafficking and downregulation of fibroblast-secreted matrix-degrading enzymes in the gut [35, 36]. Mutations in IL-21 or IL-21R seem to work differently in humans, where severe immune dysregulation characterized by infectious susceptibility to opportunistic pathogens and, paradoxically, autoimmunity has been reported in a few cases [37–39]. The pleiotropic effect of IL-21 is also reflected in a range of clinical trials where IL-21 showed immune stimulatory effects, acceptable toxicity, and antitumour effects in a fraction of cancer patients [40]. Anti-IL-21 mAb treatment has been evaluated in rheumatoid arthritis, systemic lupus erythematosus, and CD [11]. More recently, the role of anti-TNF*α* mAb in regulating IL-21 expression and Th17 cell infiltration in the intestinal mucosa of CD patients was explored [41]. IL-21 and Th17 cells were found to be highly expressed in the inflamed mucosa of active CD patients compared with healthy controls. Ten weeks after anti-TNF*α* mAb infusion, intestinal mucosal healing was improved in CD patients and IL-21 expression and Th17 cell infiltration were found to be significantly decreased [41]. Similarly, IL-21 transcripts in the intestinal tissue from CD patients have been reported to be significantly downregulated in anti-TNF*α* responders, but not in nonresponders [42]. Thus, IL-21 seems to follow CD pathogenesis and could be a potential target in both anti-TNF*α* mAb responders and nonresponders.

We demonstrate that in human intestinal material IL-21 and IL-21R expression follows the hallmark of CD, the transmural inflammation, and that IL-21R is expressed by subsets of T cells, B cells, and plasma cells located in particular germinal centers (GCs) of lymphoid aggregates and in those infiltrating the intestinal wall in general.

We also demonstrate that IL-21 neutralization only is efficacious in models with CD4 T cell-driven colitis and primarily affects homing of calprotectin-positive cells.

## 2. Materials and Methods

### 2.1. Bioethics

All human materials were obtained with informed consent from the donors/or close relatives and approval from relevant local ethical committees (H-KF-2007-0048, Rigshospitalet, Copenhagen, Denmark; Cambridge Biosciences, Supplier information: Tissue Supply Network (http://www.bioscience.co.uk)).

All mouse experiments were carried out in accordance with the European Communities Council Directive 2010/63/EU for the protection of animals used for experimental purposes and approved by the Danish Animal Experiments Inspectorate, Ministry of Food, Agriculture and Fisheries, Denmark, as well as the Internal Ethical Review Committee at Novo Nordisk A/S. Mice were sacrificed by cervical dislocation if their weight loss exceeded 20% within the study or if they had a morbid appearance.

### 2.2. Human Material

Human tissue samples used for either in situ hybridization (ISH) or immunohistochemical (IHC) investigations of IL-21 and IL-21R mRNA or protein expression are listed in Table 1.

### 2.3. Sectioning

Frozen cell and tissue sections were cut at a nominal thickness of 5 *μ*m mounted on Superfrost® Plus microscope slides, air dried, and stored at −80°C until further processing. Fixed paraffin-embedded cell and tissue sections were cut at a nominal thickness of 3 *μ*m, mounted on Superfrost Plus microscope slides, and stored at −20°C until further processing.

### 2.4. Probes

Human IL-21 (ENSG00000138684) and IL-21R (AC HS02517) cDNA fragments (344–833 bp and 725–1183 bp, resp.) were labelled with ^35^S as previously described [43, 44].

### 2.5. Antibodies for Human Study

Antibodies and conjugated detection kits used for immunohistochemical studies are listed in Supplementary Table 1. Approximately, 100 different anti-human IL-21 antibodies (commercially available and in-house produced) were evaluated on human IL-21 or mock transfected cells and positive control tissue (tonsil). One was found to be specific for recombinant human IL-21 NNC0114-0000-0032.

### 2.6. In Situ Hybridization on Human Tissue

ISH was performed on frozen sections as previously described with minor modifications [43, 44]. The protocol is described in S1.

### 2.7. Immunohistochemistry on Human Tissue

The sections were deparaffinised in xylene and rehydrated in decreasing concentrations of alcohols. Antigen retrieval was performed in Tris-EGTA buffer (10 mM; 0.5 mM), pH 9.0 in a microwave oven for 15 min. Endogenous peroxidase activity was blocked with 3% H_2_O_2_ and dual block (Dako, S2003). Endogenous biotin was blocked by incubation with avidin and biotin blocking solutions for 10 min, according to the manufacturer. Nonspecific binding was blocked by incubation with TBS containing 3% skimmed milk, 7% donkey serum, 3% human serum, and 3.2 mg/ml poly-L-lysine (PLL) for 30 min. The primary and secondary antibodies were diluted in a Tris buffer containing 0.5% skimmed milk, 7% donkey serum, 3% human serum, and 3.2 mg/ml PLL, and incubation was performed overnight at 4°C and 60 min at room temperature, respectively. The first amplification step was performed by incubation with Vectastain ABC peroxidase kit, diluted in 0.1 M Tris-HCl buffer (pH 7.5) containing 0.5% Du Pont blocking reagent (TNB) for 30 min, followed by a second amplification step with incubation in biotinylated tyramide for 6 min. The final amplification was performed by an additional incubation with the Vectastain ABC peroxidase kit, diluted as previously described for 30 min. The chromogenic reaction was achieved with diaminobenzidine. Nuclei were counterstained with haematoxylin, and the sections were rehydrated, cleared in xylene, and mounted with Pertex. The evaluation of the slides was performed by the blinded observer. The sections were evaluated by virtual microscopy using a Hamamatsu NanoZoomer (Hamamatsu Denmark, Ballerup, Denmark) and conventional microscopy using an Olympus BX51 microscope equipped with a DP70 digital camera (Olympus Denmark A/S, Ballerup, Denmark).

### 2.8. Double Immunofluorescence on Human Tissue

The immunohistochemical demonstration of the IL-21R was in general performed as described above. Upon the second amplification with biotinylated tyramide for 3 min, sections were incubated with the Vectastain ABC peroxidase kit, diluted as previously described for 30 min. The final amplification was performed by incubation with Alexa-594 conjugated tyramide diluted according to the manufacturer for 6 min. The sections were then subjected to second rounds of microwave oven treatment in Tris-EGTA buffer (10 mM; 0.5 mM), pH 9.0 for 10 min, blocking of endogenous peroxidase activity with 3% H_2_O_2_, and endogenous biotin by incubation with avidin and biotin blocking solutions for 10 min, respectively, according to the manufacturer. Additional blocking of nonspecific binding was performed by incubation with TBS containing 3% skimmed milk, 7% donkey serum, and 3% human serum for 30 min. The second primary and secondary antibodies were diluted in a Tris buffer containing 0.5% skimmed milk, 7% donkey serum, and 3% human serum, and incubation was performed overnight at 4°C and 60 min at room temperature, respectively. The final amplifications were performed by an additional incubation with the Vectastain ABC peroxidase kit, diluted as previously described for 30 min, and incubation with Alexa-488 conjugated tyramide. The nuclei were counterstained with Hoechst for 15 min, and the sections were finally mounted in fluorescence mounting media (Dako). The sections were evaluated by virtual microscopy using a Hamamatsu NanoZoomer (Hamamatsu Denmark, Ballerup, Denmark) and an FV10i Laser scanning microscope (Olympus Denmark A/S, Ballerup, Denmark).

### 2.9. Semiquantitative Scoring on Human Tissue

IL-21R immunopositive (IL-21R^+^) cells were semiquantitatively scored as follows: The mucosa-associated lymphoid compartments were individually scored: mucosa (M): intraepithelial lymphocyte (IEL) compartment (surface epithelium), lamina propria, and follicle-associated epithelium (FAE); submucosa (SM): isolated (solitary) lymphoid follicles (ILF), Peyer's patches (ileum)/colonic IEL (colon), and isolated infiltrating lymphocytes; and muscularis externa (ME): IELs and isolated infiltrating lymphocytes. Each compartment was scored on a scale from 0 to 4: 0, no; 1, few; 2 moderate; 3, many; and 4, abundant numbers of IL-21R^+^ cells. An accumulated score was calculated for each intestinal layer (M, SM, and ME) and in total (M + SM + ME) for the entire intestine: max score—M = 12, SM = 12, and ME = 8 and entire intestine = 32.

### 2.10. Mice and Housing

B6.129P2-*Il10*138 *tm1Cgn*/J (IL-10^−/−^) mice and C57BL/6J wild-type (WT) mice were obtained from Charles River Laboratories (Sulzfeld, Germany) in accordance with a licence agreement with MCG (Munich, Germany). C.B-17 severe combined immunodeficiency (SCID) mice, BALB/c, NMRI, C57BL/6NTac, B6.129.S6Rag2tm1FwaN12 mice, IL-21R^−/−^C57BL/6, and IL-21R^+/+^C57BL/6 littermates were purchased from Taconic M&B (Ry, Denmark). Female mice, 8–12 weeks old, were used in all the studies. The mice were housed at Novo Nordisk A/S, Maaloev, Denmark, and kept under barrier-protected conditions free of agents listed in FELASA guidelines [45] as follows: 10–12 mice per cage and a 12-hour light/dark cycle. 90 percent of the cage bedding was changed weekly, and 10 percent of the cage bedding was transferred between cages in order to ensure a homogenous microbial environment. The clinical status of the mice was evaluated three times weekly by visual inspection, percentage weight change, and faecal consistency.

### 2.11. Induction of Adoptive Transfer Colitis Using CD4^+^CD25^−^ T Cells

The transfer colitis with CD4^+^CD25^−^ T cells was conducted as previously described [46, 47].

The protocol is described in S1 and S6.

### 2.12. Induction of Adoptive Transfer Colitis Using CD4^+^CD25^−^CD45RB^high^ T Cells

The protocol is described in S1 and S6.

### 2.13. Induction of Piroxicam-Accelerated Colitis (PAC) in IL-10^−/−^ Mice

Piroxicam-accelerated colitis (PAC) was performed as previously described [48, 49].

The protocol is described in S1 and S6.

### 2.14. Induction of DSS Colitis

Induction of dextran sulphate sodium (DSS) colitis was performed as previously described [50, 51]. The protocol is described in S1 and S6.

### 2.15. Monitoring of Disease in Mice

Body weight was monitored three times weekly, and the mice were sacrificed if they lost more than 20% of their initial body weight. Faecal consistency was evaluated before the start of the treatment and subsequently 3 times a week using a semiquantitative score (normal stool = 0; slightly soft = 1; soft but formed = 2; not formed = 3; and liquid stools or no faeces in colon at sacrifice = 4). Disease activity index (DAI) score was calculated as previously described [48, 49, 51].

### 2.16. Collection of Serum and WBC Counts from Mice

During the experiment, the mice were anesthetized with isoflurane, and blood from the periorbital venous plexus was collected in EDTA-containing tubes for white blood cell (WBC) counts, FACS analysis, and exposure. The number of WBC per liter was analysed in samples (20 *μ*l) of EDTA-stabilized peripheral whole blood, using a Medonic CA 620 (Boule Nordic, Denmark) blood analysis apparatus according to the manufacturer's instructions. Serum samples were collected directly in microtainer SST tubes (BD, US). The isolated serum samples were then transferred to micronic tubes and stored at −80°C.

### 2.17. Flow Cytometry of Blood Samples from Mice

The flow cytometry analysis of mouse blood samples was performed as described in S1 and S6.

### 2.18. Exposure and Pharmacokinetics in Mice

Exposure and pharmacokinetic analysis was performed as described in S1 and S6.

### 2.19. Endoscopic Analysis on Mice

Mice were anaesthetized with isoflurane and placed on the back. A rigid telescope (HOPKINS straight forward, 0°) was connected to a light source/air pump (Xenon 175) and a camera (Telecam SL) all purchased from Karl Storz (Tuttlingen, Germany), as well as a monitor (Sony Triniton) and a video recorder (SCB aida DVD, Karl Storz) as described by Becker et al. [52]. The endoscope was coated with a lubricant containing lidocaine hydrochloride (Farco-pharma, Köln, Germany) and introduced via the anus into the distal 4 cm of the colon. The evaluation of the colonoscopy findings was done by two blinded observers using the Murine endoscopic index of colitis severity (MEICS) score as previously described in [52].

### 2.20. Post Mortem Analyses on Mouse Tissue

Mice were sacrificed by cervical dislocation, and the caecum, colon, and rectum were excised. The length of the colon was measured from the caeco-colonic junction to the anus. The colon was rinsed with PBS and weighed. The colonic weight : length (*W* : *L*) ratio (cm/g) was used as a macroscopic, objective parameter to verify the presence of established colitis, since it is known to correlate with the histology score [48]. The proximal 1/3 of the colon was removed, and the remaining 2/3 of the colon was bisected or trisected longitudinally. The dissected colon biopsies were processed for histological analysis, qPCR, and cytokine/chemokine profiling.

### 2.21. Histological Analysis on Mouse Tissue

Histological analyses on mouse tissues were performed as described in details in S1 and S6.

The severity of the histopathological lesions of colon segments was examined in a blinded manner, using the criteria previously described in [46, 48, 51, 53].

### 2.22. IL-21 Intracellular Cytokine Staining of Mesenteric Lymph Node (mesLN) CD4^+^ T Cells

On day 7, 16, 29, and 38 post cell transfer, mesenteric lymph node (mesLNs) and colons were aseptically removed from 5 mice per time point. Three (3) naïve BALB/c mice were included as controls. Restimulation mesLN lymphocytes or LPLs were stimulated for 4 h at 37°C/5% CO_2_ with PMA/ionomycin *in vitro* in the presence of GolgiSTOP (w/monensin) and GolgiPLUG (w/Brefeldin A). Following stimulation, cells were surface stained with murine Fc Block (*α*-CD16/32) followed by surface staining with the antibodies indicated in the following: anti-mouse CD45 Qdot605, anti-mouse TCRb FITC, anti-mouse CD4 PE, and anti-mouse CD8 Pacific Blue, NEAR-IR (APC-Cy7). Subsequently, the cells were washed and fixed in 4% PFA. The fixed cells were permeabilized by Perm/Wash buffer. Hereafter, either anti-mouse IL-21 or mouse IgG1 conjugated to AlexaFlour647 was added.

### 2.23. *In Vitro* Stimulation of mesLN CD4^+^ T Cells with IL-21

mesLN was aseptically removed from mice with AdTr and passed through a 70 *μ*m cell strainer to obtain a single-cell suspension. CD4^+^ T cells were obtained using a murine CD4-negative section kit (Miltenyi Biotec Norden, Copenhagen, Denmark). The resulting cells were cultured for 72 h in RPMI medium 1640 with FBS (10%) and penicillin/streptomycin (1%) in the presence of immobilized anti-CD3 (0–25 ng/ml) (clone 145-2c11; eBioscience, San Diego, CA, USA) and rmIL-21 (0–50 ng/ml; R&D Systems, Abingdon, UK) as well as recombinant mouse anti-mouse IL-21s mAb or mouse IgG1 (both 5 *μ*g/ml). Supernatants were subsequently collected for the analysis by a premixed mouse cytokine/chemokine multiplex antibody detection system (Milliplex, Millipore, and Billerica, MA, USA).

### 2.24. Statistical Analyses

#### 2.24.1. Human Study

The semiquantitative scoring of the immunohistochemical data for IL-21R protein expression was analysed by Mann–Whitney's two-tailed nonparametric *t*-test in GraphPad Prism 5, and *p* < 0.05 was considered significant.

#### 2.24.2. Experimental Mouse Studies

In the experimental colitis studies, GraphPad Prism version 6.0 was used for all statistical analyses. The statistical significance of differences between normally distributed, parametric data from the two groups was evaluated using unpaired Student's *t*-test. The comparison of nonparametric, non-Gaussian distributed data from the two groups was performed using a nonparametric Mann–Whitney *U* test. The statistical significance between more than two groups was evaluated by a one-way ANOVA or Kruskal–Wallis test with Holm-Sidak's or Dunn's multiple comparison test, respectively. Prophylactic treatment studies were evaluated by two-sided tests, whereas therapeutic studies were evaluated by one-sided tests.

## 3. Results

IL-21 and IL-21R mRNA and protein expression analyses were assessed on frozen OCT-embedded and fixed paraffin-embedded intestinal samples from non-IBD controls histopathologically within normal limits and from patients with CD, respectively. The intestinal types included in each study are listed in Table 1.

### 3.1. IL-21 and IL-21R Expression in the Intestine from Patients with CD

**Figure 1:**
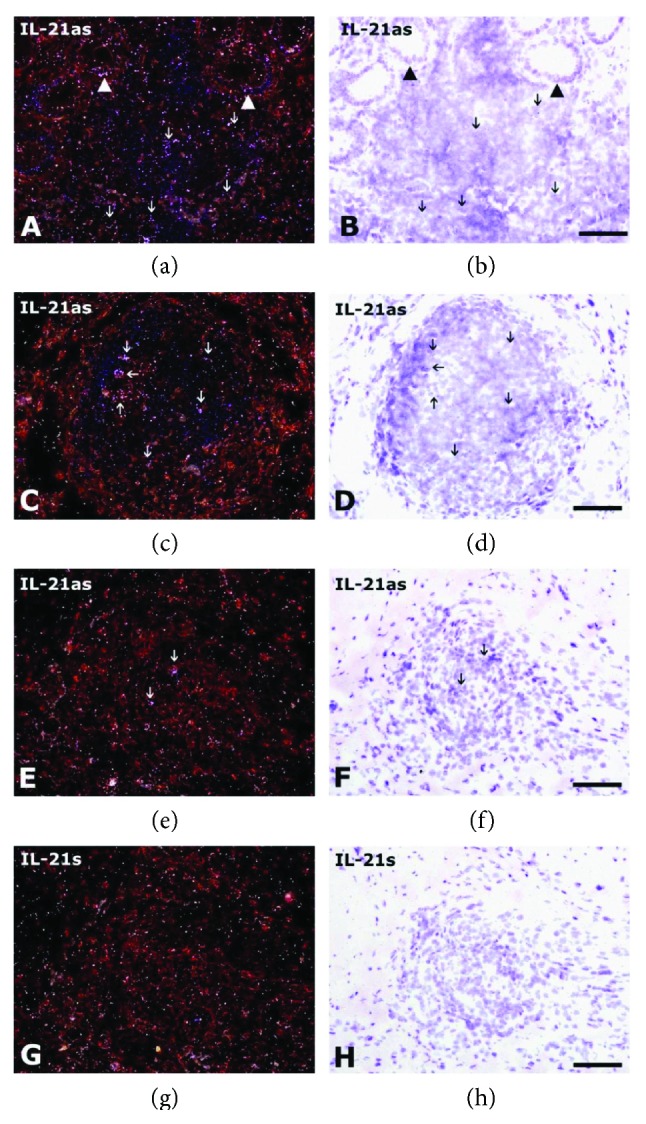
IL-21 mRNA expression in the small intestine from patients with Crohn's disease. In situ hybridization for IL-21 mRNA expression using the IL-21as (a, b, c, d, e, f) and IL-21s (g, h) probes on the small intestine from patients with CD (patient 1 (a–d), patient 2 (e-f)). IL-21 mRNA expressions are seen as white and black granules, in few isolated cells within the IEL compartment in (a) and (b) (white and black arrowheads, resp.). IL-21 mRNA-expressing cells are seen as white and black granules in lymphoid aggregates in the mucosa (white and black arrows in (a) and (b), resp.), in isolated lymphoid follicles in the submucosa (white and black arrows in (c) and (d), resp.), and in the muscularis externa (white and black arrows in (e) and (f), resp.). No specific reaction was observed with the IL-21s probe (g, h). The sections were counterstained with haematoxylin/eosin. Bars: 50 *μ*m (a–e).

**Figure 2:**
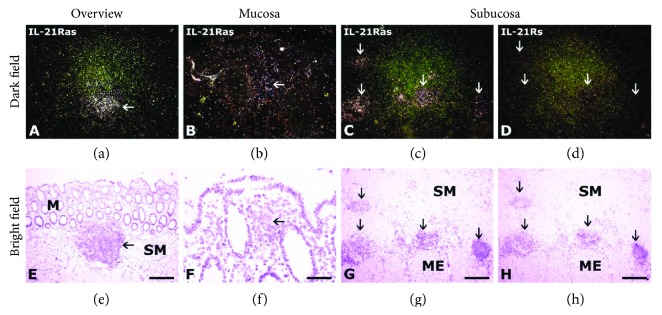
IL-21R mRNA expression in the colon from a patient with Crohn's disease. IL-21R mRNA expression was assessed by in situ hybridization using the IL-21Ras (a, b, c, e, f, g) and IL-21Rs (d, h) probes on intestines from patients with CD. Colonic IL-21R mRNA-expressing cells are seen as white and black granules in an isolated lymphoid aggregate in the submucosa (white arrow and black arrow in (a) and (e), resp.). IL-21R mRNA expression is seen in the isolated follicle-associated epithelium of the mucosa (white arrow and black arrow in (b) and (f), resp.) and in isolated lymphoid follicles in the submucosa penetrating into the muscularis externa (white and black arrows in c and g, resp.). No specific reaction was observed with the IL-21Rs probe (d, h). The sections were counterstained with haematoxylin/eosin. M: mucosa; ME: muscularis externa; SM: submucosa. Bars: 200 *μ*m (a, c, d, e, g, h) and 50 *μ*m (b, f).

As summarized in Table 1, IL-21 and IL-21R mRNA and protein-expressing cells were observed in the mucosa and submucosa from non-IBD intestinal samples within normal limits. In patients with CD, the IL-21 and IL-21R mRNA-expressing cells were generally observed as solitary cells in the lamina propria (Figures 1(a) and 1(b) and Figures 2(a), 2(b), 2(e), and 2(f), resp.), in the follicle-associated epithelium (FAE) and isolated (solitary) lymphoid follicles (ILF), Peyer's patches (ileum), and colonic-isolated lymphoid follicles (colon) of the submucosa (Figures 1(c) and 1(d) and Figures 2(c) and 2(g), resp.), and in lymphoid aggregates of the muscularis externa (Figures 1(e) and 1(f)). No reactivity was observed with the IL-21 and IL-21R sense probes (Figures 1(g) and 1(h) and Figures 2(d) and 2(h), resp.). IL-21R mRNA expression in the intraepithelial lymphocyte (IEL) compartment (e.g., surface epithelium including intraepithelial lymphocytes) could not be clearly demonstrated.

**Figure 3:**
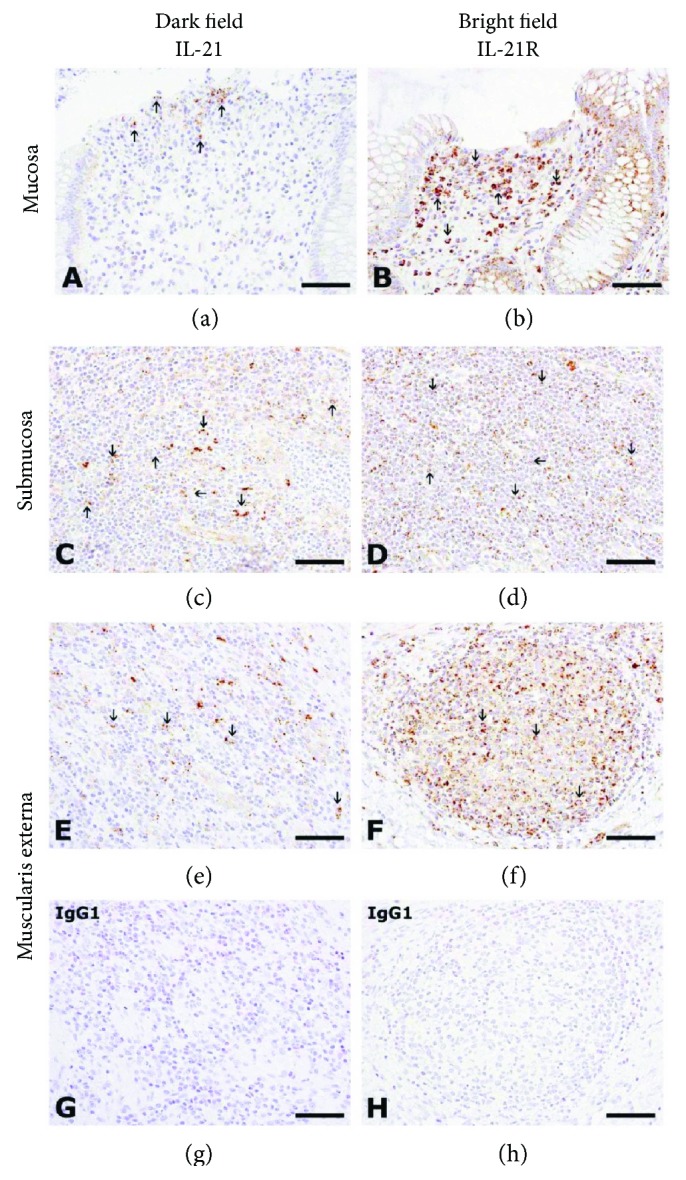
IL-21 and IL-21R protein expression in the intestine from patients with Crohn's disease. Immunohistochemical staining of IL-21 (a, c, e) and IL-21R (b, d, f) in the colon from patients with CD. IgG1 isotype-specific control antibody is shown in (g) and (h). IL-21^+^ cells (arrows in (a), (c), and (e)) and IL-21R^+^ cells (arrows in (b), (d), and (f)) are seen as solitary cells in the lamina propria of the mucosa (a, b), lymphoid aggregates of the submucosa (c and d), and muscularis externa (e, f), respectively. No reaction was observed with the IgG1 control antibody (g, h). Nuclei (in blue) were counterstained with haematoxylin. Bars: 50 *μ*m (a–h).

As summarized in Table 2, IL-21 and IL-21R immunopositive immune cells were present in intestinal non-IBD samples within normal limits in the mucosa and submucosa. In patients with CD, additional expression was observed within lymphoid aggregates of the submucosa and in the muscularis externa, with abundant expression in GCs. Thus, the general compartmentalization of IL-21 and IL-21R immunopositive cells resembled that described for IL-21 and IL-21R mRNA expressing cells, that is, within the IEL compartment, in follicle-associated epithelium and as solitary cells in the lamina propria of the mucosa (Figures 3(a) and 3(b)), in Peyer's patches/colonic-isolated lymphoid follicles and solitary cells of the submucosa (Figures 3(c) and 3(d)), and in lymphoid aggregates of the muscularis externa (Figures 3(e) and 3(f)). Finally, IL-21R immunopositive cells were generally more abundantly present than IL-21 immunopositive cells. These IL-21 immunopositive cells were present within the same areas as IL-21R immunopositive cells throughout the different immune compartments of the intestine from patients with CD. No immunoreactivity was observed with the isotype-specific control antibody (Figures 3(g) and 3(h)).

The semiquantitative analysis was performed on mucosa-associated lymphoid tissues identified according to the revised nomenclature assigned by Brandtzaeg et al. [54] and semiquantitatively scored as described in Materials and Methods.

**Figure 4:**
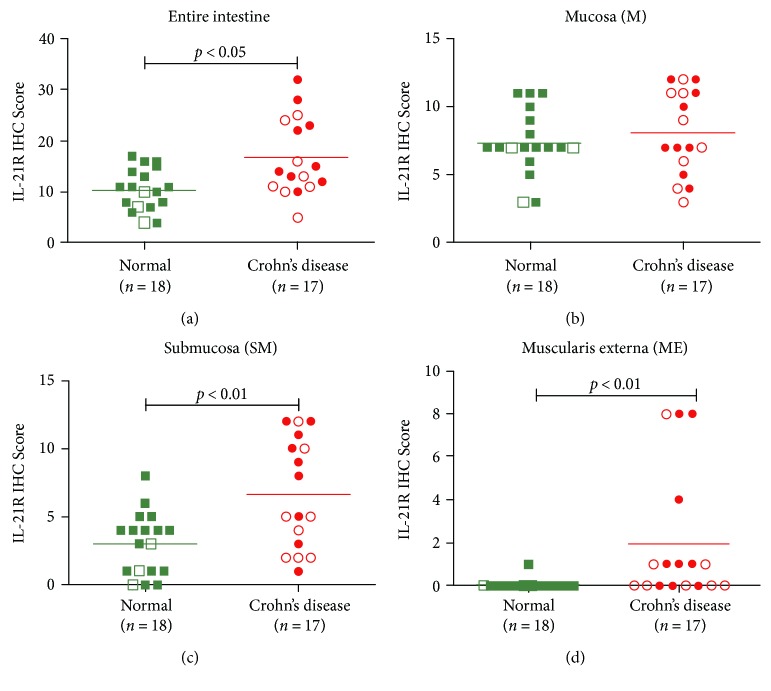
Semiquantitative analysis of the IL-21R protein expression in Crohn's disease. IL-21R protein expression was investigated by immunohistochemistry. The mucosa-associated lymphoid compartments were individually scored. Mucosa (M): intraepithelial lymphocyte compartment (surface epithelium), lamina propria, and follicle-associated epithelium. Submucosa (SM): isolated (solitary) lymphoid follicles (IEL), Peyer's patches (ileum)/colonic IEL (colon), and isolated infiltrating lymphocytes. Muscularis externa (ME): IELs and isolated infiltrating lymphocytes. Each compartment was scored on a scale from 0 to 4: 0, no; 1, few; 2 moderate; 3, many; and 4, abundant numbers of IL-21R^+^ cells. An accumulated score was calculated for each intestinal layer (M, SM, ME) and in total (M + SM + ME) for the entire intestine. Max score: M and SM = 12; ME = 8; M + SM + ME = 32. Green closed squares and red closed circles represent colon samples, whereas green open squares and red open circles represent small intestinal samples, from normal non-IBD controls and patients with CD, respectively. The data were analysed by Mann–Whitney's two-tailed nonparametric *t*-test in GraphPad Prism 5, and *p* < 0.05 was considered significant.

Based on the semiquantitative score the IL-21R was significantly highly expressed (*p* < 0.05) in the intestine from patients with CD compared to non-IBD control intestinal samples within normal limits (Figure 4(a)). The IL-21R expression did not reach significance in the mucosa (*p* = 0.47) (Figure 4(b)), but did show a significant difference in the submucosa (*p* < 0.01) (Figure 4(c)) and muscularis externa (*p* < 0.01) (Figure 4(d)).

There was no significant difference in the IL-21R expression in the IEL compartment in samples from patients with CD compared to non-IBD controls (data not shown). The study on IL-21 protein expression was not powered to perform a semiquantitative analysis of IL-21 expression.

### 3.2. Characterization of IL-21R-Positive Cells in the Intestine from Patients with Crohn's Disease

Double immunostaining for the characterization of IL-21R immunopositive (IL-21R^+^) cells in the intestine from non-IBD controls and patients with CD was performed with markers for T cells (CD3), B cells (CD20), plasma cells (CD138), or macrophages (CD68).

**Figure 5:**
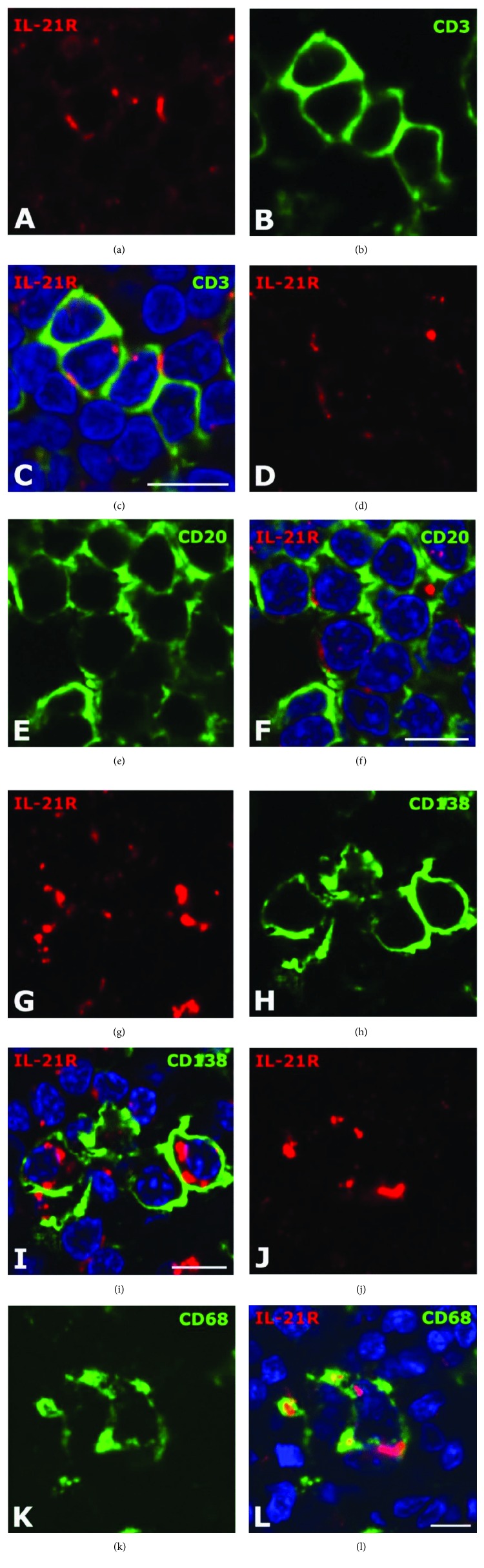
The IL-21R is expressed by T cells, B cells, plasma cells, and macrophages in the intestine from patients with Crohn's disease. Double immunofluorescence staining for the IL-21R (red signal in (a), (d), (g), and (j)), CD3^+^ T cells (green signal in (b)), CD20^+^ B cells (green signal in (e)), CD138^+^ plasma cells (green signal in (h)), and CD68^+^ macrophages (green signal in (k)) of the intestine from patients with CD analysed by confocal microscopy. Merged images of (a, b), (d, e), (g, h), and (j, k) are seen in (c), (f), (i), and (l), respectively. IL-21R^+^ T cells, plasma cells, and macrophages are seen in lymphoid aggregates of the submucosa, and IL-21R^+^ B cells are seen in the lymphoid aggregate of the muscularis externa. The nuclei (in blue) were counterstained with Hoechst. Bars: 10 *μ*m (c, f, i, l).

IL-21R^+^CD3^+^ T cells were present in the mucosa, submucosa (Figures 5(a)–5(c)), and muscularis externa, interspersed in general in the different intestinal layers and in particular within lymphoid aggregates. Likewise, IL-21R^+^CD20^+^ B cells were found in the mucosa, lymphoid aggregates, and particularly GCs of the submucosa and muscularis externa from patients with CD (Figures 5(d)–5(f)). In addition, IL-21R^+^CD138^+^ plasma cells were observed only in the mucosa in the normal intestine (data not shown), whereas CD IL-21R^+^ plasma cells were also present in lymphoid aggregates of the submucosa (Figures 5(g)–5(i)) and muscularis externa. Finally, IL-21R^+^CD68^+^ macrophages were present in the mucosa and lymphoid aggregates of the submucosa (Figures 5(j)–5(l)) and muscularis externa from patients with CD as well as in non-IBD controls (data not shown). The study was not powered to perform a quantitative analysis of the individual subsets of IL-21R^+^ cells.

### 3.3. IL-21-Producing CD4^+^ T Cells Are Upregulated in the CD4^+^CD25^−^ T Cell Adoptive Transfer (AdTr) Colitis Model

Because IL-21 is reported to be produced by both human and mouse Th17 cells [6, 24] and since Th17 cells have been shown to be involved in adoptive transfer colitis, we initially focused on the CD4^+^CD25^−^ T cell transfer model of colitis. Moreover, before treatment, studies were initiated; target validation was conducted to describe the dynamics of IL-21 protein expression by CD4^+^ T cells during the development of colitis.

**Figure 6:**
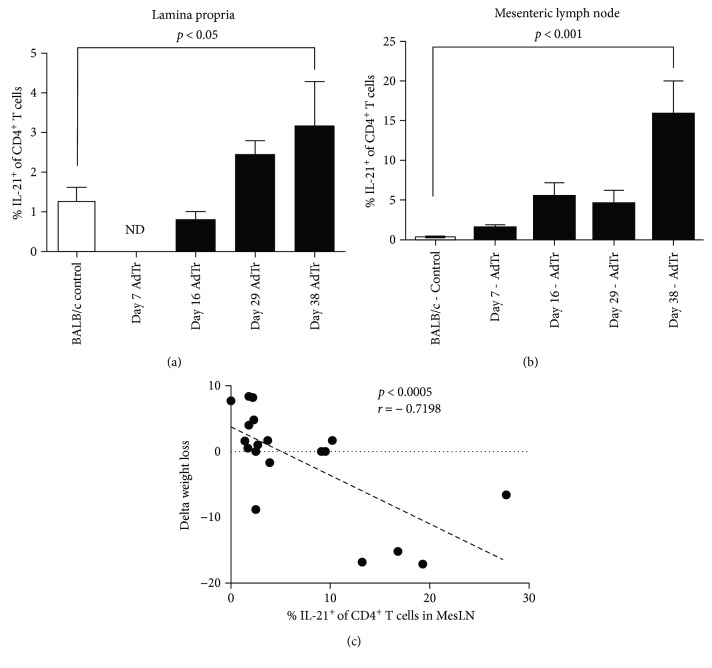
IL-21 expression is upregulated in mesenteric lymph node and lamina propria CD4^+^ T cells from mice with AdTr colitis. Frequency of CD4^+^IL-21^+^-positive T cells in the lamina propria on days 7, 17, 29, and 38 versus BALB/c controls (a). Frequency of CD4^+^IL-21^+^-positive T cells in mesenteric lymph nodes on days 7, 17, 29, and 38 versus BALB/c controls (b). Frequency of CD4^+^IL-21^+^-positive T cells versus delta weight loss in mice with AdTr (c).

The percentage of IL-21^+^CD4^+^ T cells was shown to increase over time, in both the mesLN and the lamina propria (Figures 6(a) and 6(b)). As well, the percentage of mesLN IL-21^+^CD4^+^ cells correlated with the degree of body weight change in individual mice with colitis, indicating that IL-21 may be involved in colonic disease development in this model (Figure 6(c)).

### 3.4. A Mouse Anti-Mouse IL-21 mAb Inhibits IL-21-Driven Cytokine and Chemokine Production from mesLN CD4^+^ T Cells

**Figure 7:**
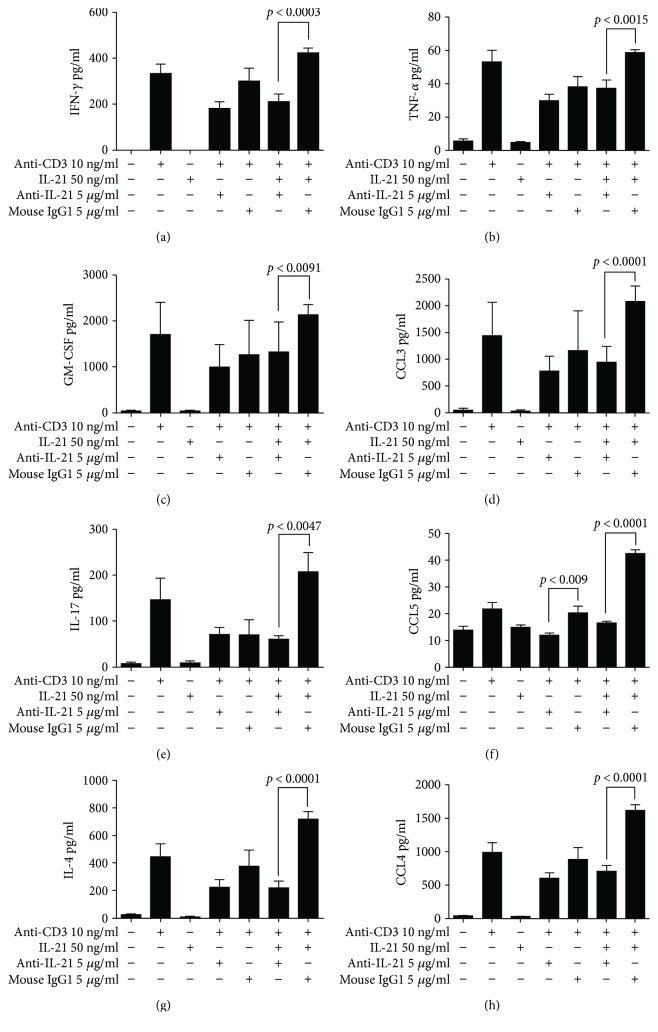
Cytokine and chemokine secretion from mesenteric lymph CD4^+^ T cells stimulated with anti-CD3 and/or IL-21. CD4^+^ T cells were isolated from mesenteric lymph nodes from mice with AdTr colitis. IFN-*γ* (a), TNF-*α* (b), GM-CSF (c), CCL3 (d), IL-17 (e), CCL5 (f), IL-4 (g), and CCL4 (h) secretion from *in vitro* stimulated CD4^+^ T cells. Cells were stimulated with plate bound anti-CD3 and/or IL-21 in combination with anti-IL-21 or isotype control (mouse IgG1).

Next, we tested whether a neutralizing mouse anti-mouse IL-21 monoclonal antibody (mAb) could neutralize anti-CD3 or anti-CD3/IL-21-induced production of proinflammatory cytokines and chemokines from mesLN CD4^+^ T cells isolated from mice with colitis (Figures 7(a)–7(g)). Neutralization of endogenous IL-21 only induced minor decreases in cytokine and chemokine production followinganti-CD3 stimulation. The only significantly regulated chemokine was CCL5 (*p* < 0.009) (Figure 7(f)). When exogenous IL-21 was added, the effect of the blocking anti-IL-21 mAb was much more pronounced with a significant inhibition of IFN-*γ*, TNF-*α*, GM-CSF, CCL3, IL-17, CCL5, IL-4, and CCL-4 production (Figures 7(a)–7(g)). No significant effect was seen on IL-2, IL-5, and IL-6 (data not shown).

### 3.5. Pharmacokinetics of Anti-IL-21 mAb in Mice

In a pilot study, we determined the exposure and the pharmacokinetics of our mouse anti-IL-21 mAb and performed modelling using a 1-compartment model. Exposure from AdTr colitis mice using two selected doses of mouse anti-mouse IL-21 (3.3 mg/kg and 25 mg/kg) was compared with the simulated exposure levels based on pharmacokinetics in healthy NMRI mice. At both doses (3.3 and 25 mg/kg), circulating unbound mouse anti-IL-21 mAb was detected (Figure S1) and matched the simulated exposure. The 25 mg/kg dose interval was selected in subsequent studies in order to have the highest possible peripheral exposure.

### 3.6. Prophylactic Treatment with Anti-IL-21 mAb Ameliorates CD4^+^CD25^−^ T Cell AdTr Colitis

Next, we wanted to assess whether treatment with 25 mg/kg anti-IL-21 mAb could prevent adoptive transfer colitis, compared to mice treated with mIgG1.

**Figure 8:**
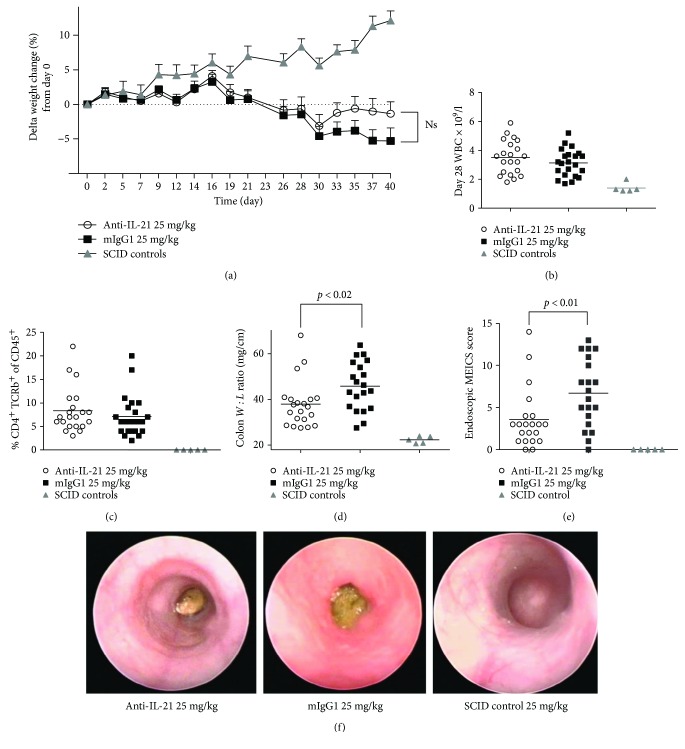
Prophylactic treatment of AdTr mice with anti-IL-21 mAb ameliorates colitis. Open circles indicate individual anti-IL-21 mAb-treated mice, black squares indicate individual mIgG1-treated mice, and grey triangles indicate individual control SCID mice. Delta body weight change over time (a). White blood cell count on day 28 (b). Frequency of CD4^+^TCRb^+^ T cell in blood on day 28 (c). Colon weight : length ratio (d). Endoscopic score encompassing (thickening of the colon, changes of vascular pattern, visible fibrin, granularity of mucosal surface, and stool consistency) on day 28 (e). Pictures showing representing images of the colon in anti-IL-21 mAb-treated mice (left), mIgG1-treated mice (middle), and SCID control mice (right) (f).

The change in body weight over the course of the experiment is shown in Figure 8(a). As previously described [46, 47], mice treated with mIgG1 started to lose body weight in the 3rd-4th week following transfer. In comparison, mice treated with anti-IL-21 mAb showed some weight loss in week 3, but stabilized at a level above the isotype control. The difference did not reach statistical significance (*P* < 0.13) at study termination.

Blood samples were obtained on day 28 in order to evaluate whether anti-IL-21 merely prevents T cell proliferation. Mice treated with anti-IL-21 mAb did not show any reduction in WBC counts compared to control treated mice (Figure 8(b)). Neither analysis of the peripheral blood did reveal any changes in CD4^+^ T cell frequencies (Figure 8(c)). In the AdTr colitis model, the disease can be associated with thickening of the colon wall. This is related to the infiltration of donor T cells and the resulting host inflammatory response. The host response includes infiltration of additional inflammatory cells (e.g., neutrophils and macrophages), vascular leakage resulting in intestinal oedema, and varying degrees of mucosal epithelial hyperplasia, all of which contribute to the thickness of the wall of the colon. In turn, there is a compensatory shortening of the colon. The ratio of colon weight (W) to length (L) provides a colon thickening index, which correlates with colitis severity and histopathology.

Mice treated with mouse anti-IL-21 mAb had a significant reduction in the colon *W* : *L* ratio, compared to mIgG1-treated mice (*P* < 0.02) (Figure 8(d)).

To acquire a translational clinical endpoint, a 2 mm endoscope was introduced via the mouse anus. Endoscopic pictures were obtained to allow the monitoring and grading of inflammation. The grading uses the murine endoscopic index of colitis severity (MEICS) score encompassing (thickening of the colon, changes of vascular pattern, visible fibrin, granularity of mucosal surface, and stool consistency). Mice treated with the anti-IL-21 mAb had a significant reduction in their endoscopic score on day 28, compared to mice treated with mIgG1 (*P* < 0.01) (Figures 8(e) and 8(f)).

**Figure 9:**
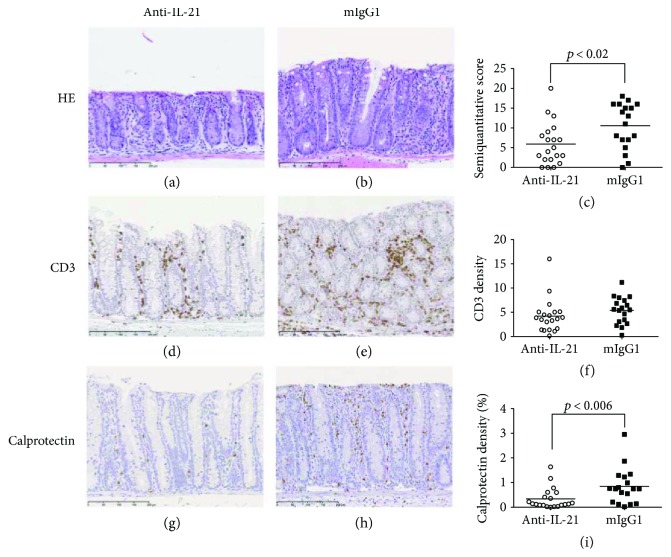
Prophylactic treatment with anti-IL-21 mAb reduces colonic transformation and neutrophil infiltration. All images are from animals representing mean values of the individual analyses. (a), (d), and (g) show the colon of AdTr mice treated with anti-IL-21 mAb, and (b), (e), and (h) show animals treated with the control mIgG1 antibody. (a, b) HE staining showing the hyperplasia of the mucosa significantly reduced (*p* < 0.02) by treatment with anti-IL-21 as seen in the histopathological analysis (c). (d, e) Immunohistochemical demonstration of CD3-positive T cells (in brown) in the mucosa and submucosa. (f) The density- (in %) positive T cells quantified by digital image analysis did not reach significance. (g, h) Immunohistochemical demonstration of calprotectin-positive neutrophils (in brown). (i) The density (in %) calprotectin-positive neutrophils and macrophages quantified by digital image analysis were significantly reduced (*p* < 0.006) by treatment with anti-IL-21. Nuclei (in blue) were counterstained with haematoxylin. Bars: 200 *μ*m (a, b, d, e, g, h).

A significant reduction in the histopathology score was also observed in mice treated with anti-IL-21 mAb compared to mIgG1 (Figures 9(a)–9(c)). The analysis of the inflammatory subsets by immunohistochemistry revealed that only calprotectin-positive cells (neutrophils and macrophages) were significantly reduced (*P* < 0.006), whereas no significant effect could be detected in the CD3 density (Figures 9(g)–9(i) and Figures 9(d)–9(f), resp.). We also evaluated changes in mRNA transcripts (Th1, Th2, Th17, and Treg) in colonic biopsies. The only transcript that was significantly downregulated following the anti-IL-21 mAb treatment was GATA3 (Supplementary Figure 2).

### 3.7. Interventive Treatment with Anti-IL-21 mAb Has a Moderate Effect on Disease Parameters in the CD4^+^CD25^−^ T Cell AdTr Model

**Figure 10:**
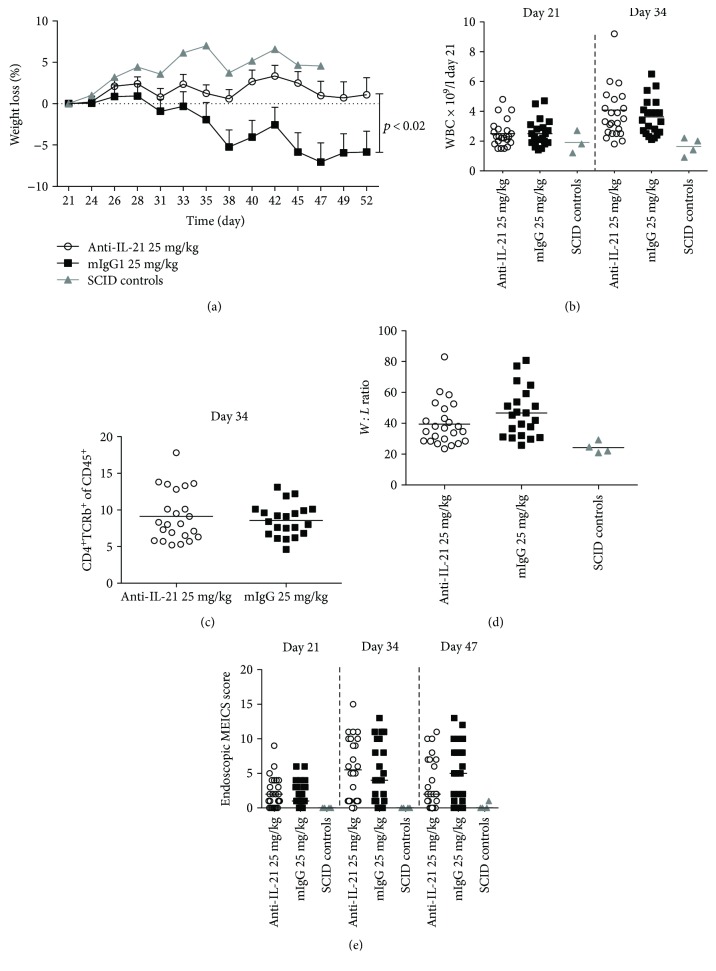
Interventive treatment of AdTr mice with anti-IL-21 mAb. Open circles indicate individual anti-IL-21 mAb-treated mice, black squares indicate individual mIgG1-treated mice, and grey triangles indicate individual control SCID mice. Delta body weight change over time (days) (a). White blood cell count on days 21 and 34 (b). Frequency of CD4^+^TCRb^+^ T cells in blood on day 34 (c). Colon weight : length ratio (d). Endoscopic score encompassing (thickening of the colon, changes of vascular pattern, visible fibrin, granularity of mucosal surface, and stool consistency) on days 21, 34, and 47 (e).

**Figure 11:**
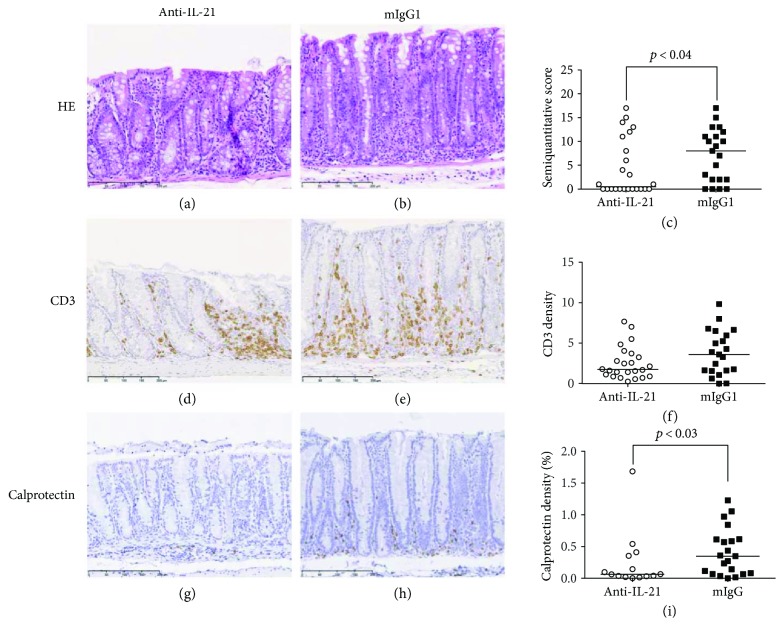
Interventive treatment with anti-IL-21 mAb reduces colonic transformation and neutrophil infiltration. All images are from animals representing mean values of the individual analyses. (a), (d), and (g) show the colon of AdTr mice treated with anti-IL-21 mAb and (b), (e), and (h) show animals treated with the control mIgG1 antibody. (a, b) HE staining showing the hyperplasia of the mucosa significantly reduced (*p* < 0.04) by treatment with anti-IL-21 as seen in the histopathological analysis (c). (d, e) Immunohistochemical demonstration of CD3-positive T cells (in brown) in the mucosa and submucosa. (f) The density- (in %) positive T cells quantified by digital image analysis did not reach significance. (g, h) Immunohistochemical demonstration of calprotectin-positive neutrophils (in brown). (i) The density (in %) calprotectin-positive neutrophils and macrophages quantified by digital image analysis were significantly reduced (*p* < 0.03) by treatment with anti-IL-21. Nuclei (in blue) were counterstained with haematoxylin. Bars: 200 *μ*m (a, b, d, e, g, h).

**Figure 12:**
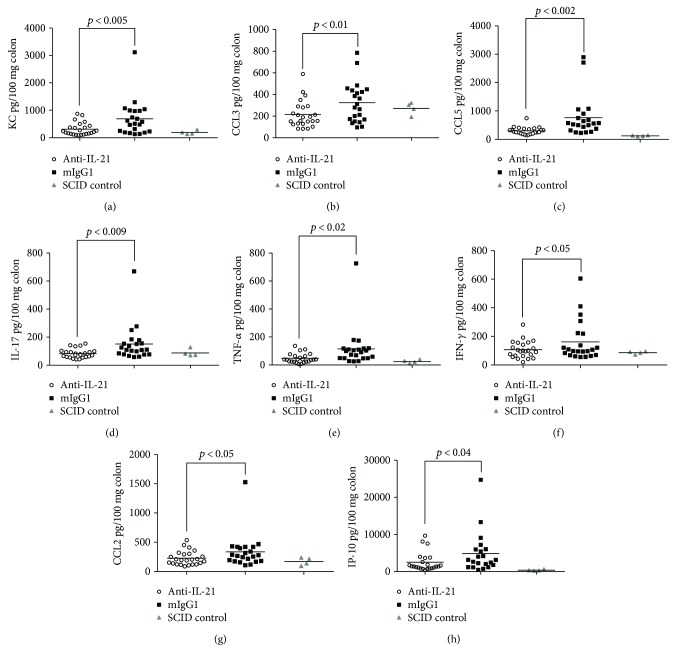
Cytokine and chemokine profile in inflamed colon from mice treated with Anti-IL-21 mAb or mIgG1. Colon biopsies isolated from individual mice with AdTr colitis. Open circles anti-IL-21 25 mg/kg, black squares mIgG1, and grey triangles SCID controls. KC (a), CCL3 (b), CCL5 (c), IL-17 (d), TNF*α* (e), IFN-*γ* (f), CCL2 (g), and IP-10 (h) were found to be significantly downregulated in anti-IL-21 mAb-treated mice.

We have previously shown that mild to moderate colonic inflammation is present at day 21 [47]. Thus, we randomized our treatment groups according to weight on day 21. Anti-IL-21 mAb treatment had a significant effect on body weight loss over the treatment period (Figure 10(a)). However, even though there was a tendency to lower colonic disease score in mice treated with anti-IL-21 mAb, no significant effect could be detected on WBC counts, CD4^+^TCR^+^ T cell frequency, colon *W* : *L* ratio, and endoscopic score (Figures 10(b)–10(e)). Colonic inflammation was further assessed by the evaluation of the histopathology score, density of CD3, and calprotectin staining. Similar to the prophylactic study, both histopathology score (Figures 11(a)–11(c)) and calprotectin density (Figures 11(g)–11(i)) were significantly reduced following anti-IL-21 mAb treatment, whereas no significant effect on CD3 density could be detected (Figures 11(d)–11(f)). Cytokine and chemokine profiling of colon biopsies from mice confirmed a significant interventive treatment effect of anti-IL-21 mAb on chemokine and Th17-related parameters (KC, CCL3, CCL5, IL-17, TNF*α*, IFN-*γ*, CCL2, and IP-10) (Figure 12). However, TNF*α*, IFN-*γ*, CCL2, and IP-10 were only borderline significant and determined by few mice with high colonic levels. Four SCID control mice were included in the analysis to show the cytokine and chemokine background level. In the isotype-treated mice all cytokines and chemokines besides CCL3 were elevated compared to SCID control mice.

No significant treatment effect was observed for IL-13, IL-10, IL-5, IL-6, IL-9, IL-12p70, IL-1*β*, IL-7, IL-2, IL-4, IL-5, and G-CSF (data not shown).

### 3.8. Ablation of IL-21 Signalling Ameliorated Colitis in the CD4^+^CD45RB^high^ Model

**Figure 13:**
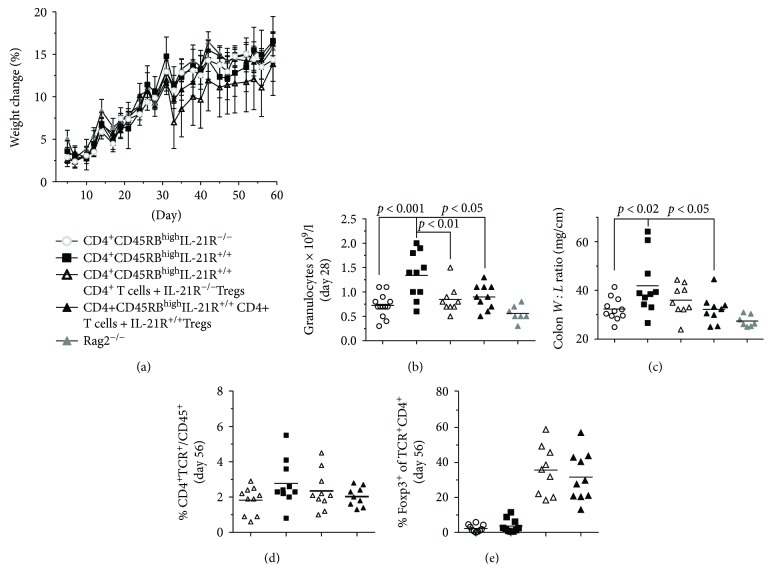
Ablation of IL-21 signalling reduces neutrophil counts and colon *W* : *L* ratio in the CD4^+^CD45RB^high^ model. Open circles indicate individual mice transferred with CD4^+^CD45RB^high^IL-21R^−/−^ CD4^+^ T cells, black squares indicate mice transferred with CD4^+^CD45RB^high^IL-21R^+/+^ CD4^+^ T cells, open triangles indicate mice transferred with CD4^+^CD45RB^high^IL-21R^+/+^ CD4^+^ T cells + IL-21R^−/−^ Tregs, black triangles indicate mice transferred with CD4^+^CD45RB^high^IL-21R^+/+^ CD4^+^ T cells + IL-21R^+/+^ Tregs, and grey triangles indicate Rag2^−/−^ control mice. Delta body weight change over time (days) (a). Granulocyte in full blood day 28 (b). Colon weight : length ratio (c). Frequency of CD4^+^TCR^+^ in full blood day 56 (d). Frequency of FoxP3^+^CD4^+^TCR^+^ in full blood day 56 (e).

Since absolute neutralization of cytokine signalling on CD4^+^ T cells may be difficult to achieve with an antibody, we tested whether naïve CD4^+^ T cells would require the IL-21R to develop chronic mucosal inflammation in the CD4^+^CD45RB^high^ model using B6.129S6-RAG2^−/−^ mice as recipients. In short, no effect was detected on body weight change, but mice transferred with CD4^+^CD45RB^high^ IL-21R^−/−^ T cells had a significantly lower colon *W* : *L* ratio as well as reduced numbers of granulocytes in the blood, compared to mice transferred with CD4^+^CD45RB^high^IL-21R^+/+^ T cells (Figures 13(a)–13(c)). Moreover, no significant difference between Tregs expressing the IL-21R and Tregs lacking the IL-21R could be detected in cotransfer experiments. However, CD4^+^IL-21R^+/+^ Tregs did reduce the colon *W* : *L* ratio significantly when compared to mice only transferred with CD4^+^CD45RB^high^IL-21R^+/+^ T cells (Figure 13(c)).

### 3.9. Prophylactic Treatment with Anti-IL-21 Has No Treatment Effect in the Piroxicam-Accelerated Colitis Mouse Model

Anti-IL-21 mAb treatment was also evaluated in the piroxicam-accelerated colitis (PAC) mouse model. This model is more acute and is not directly driven by CD4^+^ T cells [48, 49].

**Figure 14:**
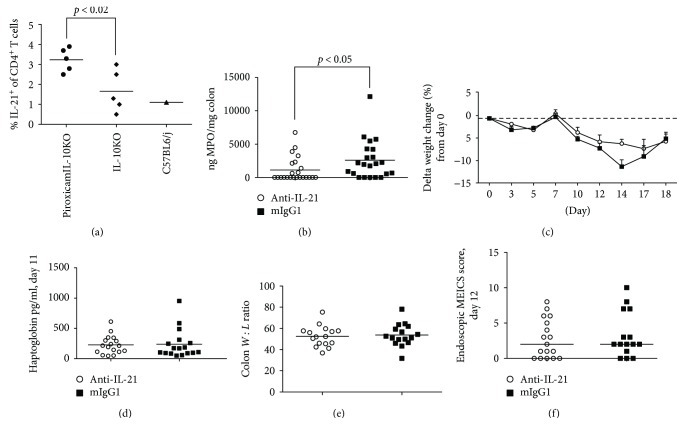
IL-21 expression and anti-IL-21 mAb treatment in piroxicam-accelerated colitis. Black circles PAC mice, black diamonds IL-10 k.o. mice, and black triangles C57BL6/j mice. Frequency of CD4^+^IL-21^+^-positive T cells in mesLN (a). Open circles anti-IL-21 25 mg/kg and black squares mIgG1 25 mg/kg. MPO levels in colonic biopsies at day 18 (b). Delta weight loss over time (days) (c). Serum haptoglobin levels on day 11 (d). Colon weight : length ratio (e). Endoscopic score encompassing (thickening of the colon, changes of vascular pattern, visible fibrin, granularity of mucosal surface, and stool consistency) on day 12 (f).

First, the frequency of CD4^+^ T cells expressing IL-21 was determined in mesLN. As seen in Figure 14(a), the percentage of IL-21^+^CD4^+^ T cells was significantly elevated in PAC IL-10 k.o. compared to IL-10 k.o. littermates. Still, the percentage of IL-21^+^CD4^+^ T cells was lower compared to the AdTr colitis model (Figure 6). Following prophylactic treatment with anti-IL-21 mAb, no significant treatment effect could be observed on systemic parameters. At day 18, the mean body weight change of anti-IL-21 mAb and mIgG1-treated mice was minus 5%, and no significant difference in weight change could be observed (Figure 14(c)). The systemic serum level of the acute phase protein (haptoglobin) level at day 11 was unchanged (Figure 14(d)). As well, anti-IL-21 mAb treatment had no effect on colonic parameters such as the colon *W* : *L* ratio at termination or endoscopic score day 12 (Figures 14(e) and 14(f)). The only indication of a positive treatment effect was a significant reduction in colonic MPO levels (Figure 14(b)). Indicating a potential effect on neutrophils and macrophages.

## 4. Discussion

IL-21 has been shown to be upregulated in the intestine from patients with IBD in several studies by different methods such as Western blotting of total protein extracts from tissue specimens, culture of lamina propria mononuclear cells (LPMCs) followed by ELISA on supernatants, intracellular flow cytometry on LPMCs [22, 55, 56], and immunohistochemistry of mucosal biopsies [57]. In the present study, using resected intestinal human samples, the presence of IL-21 mRNA and protein-expressing cells was described in different immune cell compartments in the intestine from patients with CD. IL-21 mRNA was primarily expressed by solitary cells in the IEL compartment, lamina propria, and follicle-associated epithelium of the mucosa, in isolated lymphoid follicles in the submucosa as well as in infiltrates in the muscularis externa.

Upregulation of the IL-21R in the intestine from patients with IBD has likewise been demonstrated by different groups using immunohistochemistry [35, 56]. In the present study, these data were confirmed, as the IL-21R was significantly upregulated in the intestine from patients with CD compared to non-IBD control samples. Moreover, the IL-21R mRNA expression was similar to the IL-21R protein expression pattern in the immune cell compartment characteristic of the intestinal wall. Specifically, IL-21R mRNA and protein were expressed by solitary immune cells in the lamina propria and follicle-associated epithelium of the mucosa, in isolated lymphoid follicles in submucosa, as well as in cellular infiltrates of the muscularis externa. Moreover, increased IL-21R protein expression was restricted to the submucosa and muscularis externa. Thus, IL-21 and IL-21R expression followed the histopathological hallmark of CD, that is, the transmural inflammation that was observed in the resected material investigated herein as compared to studies performed on mucosal biopsies [35, 56, 57]. Furthermore, we identified the IL-21R^+^ cells as the subsets of T cells, B cells, plasma cells, and macrophages; however, the study was not powered to detailed quantitative analyses.

In this context, one may speculate that the expression of the IL-21R on CD20^+^ B cells, CD138^+^ plasma cells, and the CD68^+^ macrophages directly and/or indirectly has an effect on T cell activation, B cell maturation, and immunoglobulin production from plasma cells. Whether a similar situation applies to patients with UC remains to be examined in full size biopsies. However, it may be hypothesized that the effect of IL-21 in patients with UC is related to the cytokine pleiotropic effect on neutrophils and/or macrophages, a hallmark for the inflammation in these patients.

Previous studies have shown that mice lacking IL-21 are unable to upregulate Th17-associated molecules during experimental colitis and are largely protected from DSS-induced colitis [31, 34, 58, 59]. Similarly, immunodeficient mice adoptively transferred with CD4^+^IL-21^−/−^ T cells develop less severe colitis [60, 61], and mice treated in a prophylactic setup with a neutralizing IL-21R/Fc fusion protein or anti-IL-21 mAb are protected from both DSS and TNBS-induced colitis [31, 58, 59]. In our AdTr study with CD4^+^CD25^−^ T cells, we observed a clear production of IL-21 from the lamina propria and mesenteric lymph node CD4^+^ T cells. Moreover, we observed a reduction of colonic inflammation following both prophylactic and interventive treatments with 25 mg/kg anti-IL-21 mAb. This treatment effect was not associated with a noticeable reduction in WBC, CD4^+^ T cell frequency, or colonic CD3-infiltrating T cells, suggesting that the effects of neutralizing IL-21 in this model may not depend on the prevention of T cell proliferation or migration. However, we observed a reduction in colonic calprotectin (marker of neutrophils), several chemokines, and proinflammatory cytokines, supporting the idea that IL-21 neutralization *in vivo* not only affects CD4^+^ T cell differentiation but also regulates neutrophil infiltration through direct or indirect mechanisms. To further dissect the importance of IL-21 signalling in innate lymphoid cells and nonimmune cells, future studies should investigate the *in vivo* effects of transferring CD4^+^ T cells to IL-21R^−/−^Rag2^−/−^ recipient mice. In our piroxicam-accelerated colitis study, where colitis symptoms are more acute and not directly driven by CD4 T cells [48, 49], we did not observe any protective effect of IL-21 neutralization. However, if CD4^+^ T cells are the primary producer of IL-21, this may not be surprising.

The underlying mechanism of IL-21 signalling in experimental colitis is still unclear. Most groups describe a downregulation of Th17 cells and associated molecules [31, 34, 58, 59], while other groups report that IL-21 is not essential for Th17 cell development [60–62].

We explored the direct effect of IL-21 signalling in CD4^+^ T cells by transferring either CD45RB^high^IL-21R^−/−^ CD4^+^ T cells or CD45RB^high^IL-21R^+/+^ CD4 T cells to Rag2 k.o. mice. This concept is clearly different from transferring CD4^+^ T cells lacking IL-21 or IL-21 neutralization by treatment, since other cell types, for example, innate lymphoid cells or fibroblasts may respond to IL-21 secreted from the CD4^+^IL-21R^−/−^ T cells. In our study, we observed less severe colitis in Rag2 k.o. mice transferred with CD45RB^high^IL-21R^−/−^ CD4^+^ T cells compared with mice transferred with CD45RB^high^IL-21R^+/+^ CD4^+^ T cells. In addition, mesLN CD4^+^ T cells isolated from mice with colitis responded with excessive production of proinflammatory cytokines and chemokines following stimulation with exogenous IL-21. The observation supports that IL-21 signalling may play a part in the initial development and differentiation of pathogenic CD4^+^ T cells. However, we could not detect a marked reduction in proinflammatory cytokine or chemokine production following in vitro neutralization of endogenous IL-21 production by mesLN CD4^+^ T cells. Thus, only high IL-21 levels seem to mediate the secretory effect. In addition, we could not detect any significant changes in Th17, Th1, or Treg transcription factors in colonic biopsies; only Th2 transcripts were downregulated by anti-IL-21 mAb treatment.

Moreover, several studies suggest that IL-21 mediates inhibitory effects of peripheral differentiation of Tregs and makes CD4^+^ T cells resistant to Tregs-mediated immune suppression [34, 63]. However, in our CD45RB^high^CD25^+^CD4^+^ T cell transfer study, only minor effects of IL-21R ablation could be detected on Treg frequency or *in vivo* suppressive function. We speculate that IL-21 in our model primarily affects the differentiation of Th17 in mesLN rather than affecting Tregs directly.

Recently, Wang et al. [64] reported that IL-21/IL-21R signalling may actually suppress intestinal inflammation induced by DSS through regulation of Th responses. Still, in most studies, IL-21^−/−^ mice have been reported to be protected from DSS-induced colitis [31, 34, 58, 59]. In our facility, IL-21R^−/−^ mice are not protected from DSS-induced colitis neither in an acute nor in a chronic setup (Supplementary Figures 3 and 4). The discrepancy between colitis studies with IL-21 or IL-21R^−/−^ mice may be due to variability in microflora between laboratories, T cell dependency in the model setup, or a consequence of transcriptional changes in the areas around the targeted gene in the k.o. mice. However, it also highlights the complex biology associated with IL-21 signalling.

IL-21 is overproduced in many chronic inflammatory disorders, and studies in experimental models indicate that IL-21 plays an important role in sustaining tissue damage. Moreover, since IL-21 is a pleiotropic cytokine and since CD most likely is caused by multiple immunological subsets, the stimulatory role of IL-21 on non-CD4^+^ T cells, for example, neutrophils, IgA-producing B cells, fibroblasts, and macrophages, should be further investigated.

## Figures and Tables

**Table 1:** Human tissues used in the present study.

Tissue	Processed			Vendor
	FF^∗^ (ISH)	FFPE^∗^ (IHC)	PFPE^∗^ (IHC)	
*Positive control tissue*				
Tonsil	3		5	Rigshospitalet (Copenhagen, DK)
*Normal non-IBD controls* ^∗∗^				
Colon	14	15		Cambridge BioScience (Cambridge, UK)
Small intestine	3	3		Cambridge BioScience (Cambridge, UK)
*Crohn's disease*				
Colon	8	10		Cambridge BioScience (Cambridge, UK)
Small intestine	7	10		Cambridge BioScience (Cambridge, UK)

FF: fresh frozen OCT embedded; FFPE: formalin-fixed paraffin embedded; IHC: immunohistochemistry; IBD: inflammatory bowel disease; ISH: in situ hybridization; PFPE: paraformaldehyde-fixed paraffin embedded. ^∗^Number of samples is listed. ^∗∗^Pathologically classified as being within normal limits obtained from patients with adenocarcinoma, gastrointestinal tumour of small intestine, lipoma, dysplasia of bile duct epithelium, and familial adenomatous polyposis.

**Table 2:** Summary of IL-21 and IL-21R expression in the intestine from patients with Crohn's disease.

Diagnosis	Target	Method	Entire intestine	Mucosa	Submucosa	Muscularis externa
Normal non-IBD controls	IL-21	ISH (mRNA)	10/15	8/15	7/15	0/15
	IL-21R	ISH (mRNA)	11/17	9/17	8/17	0/17
	IL-21	IHC (protein)	5/5	5/5	1/5	0/5
	IL-21R	IHC (protein)	18/18	18/18	15/18	1/18

Crohn's disease	IL-21	ISH (mRNA)	10/13	9/13	10/13	5/13
	IL-21R	ISH (mRNA)	12/12	11/12	8/12	2/12
	IL-21	IHC (protein)	6/6	3/6	5/6	4/6
	IL-21R	IHC (protein)	17/17	17/17	17/17	9/17

A positive patient is identified as having IL-21/IL-21R-expressing cells. Numbers of IL-21/IL-21R-positive patients/total number of patients included in the study are listed. IHC: immunohistochemistry; ISH: in situ hybridization.

## Supplementary Materials

Supplementary 1 Supplementary Table 1: materials, sera, antibodies, and detection kits.

Supplementary 2 Supplementary Figure 1: exposure from AdTr colitis mice using two selected doses of mouse anti-mouse IL-21 mAb (3.3 mg/kg and 25 mg/kg) compared with simulated exposure levels.

Supplementary 3 Supplementary Figure 2: transcriptional regulation in colonic biopsies after treatment with anti-IL-21 mAb.

Supplementary 4 Supplementary Figure 3: ablation of IL-21 signalling has no effect on acute and relapsing DSS-induced colitis. Open circles indicate IL-21R^−/−^ mice, black squares indicate IL-21R^+/+^ mice, and grey triangles indicate IL-21R^−/+^ mice.

Supplementary 5 Supplementary Figure 4: ablation of IL-21 signalling has no significant effect on chronic DSS-induced colitis. ^−/−^ mice; black squares indicate IL-21R^+/+^ mice. Colon weight : length ratio (A). Endoscopic score encompassing (thickening of the colon, changes of vascular pattern, visible fibrin, granularity of mucosal surface, stool consistency) on day 20 (B). Histopathology score (C).
