# Supplementary material for: Evaluating IL-21 as a Potential Therapeutic Target in Crohn's Disease
Source: Gastroenterol Res Pract. 2018 Apr 10;2018:5962624. doi: 10.1155/2018/5962624 (PMC5914125; doi:10.1155/2018/5962624)
Supplement: Supplementary 1 — Supplementary Table 1: materials, sera, antibodies, and detection kits. [file 5962624.f1.docx]

**Supplementary table 1 Materials, sera, antibodies and detection kits**

| **Material** | **Vendor** | **Cat. #** | **Clone** | **Isotype** |
| --- | --- | --- | --- | --- |
| **Serum** |  |  |  |  |
| Donkey | Jackson ImmunoResearch  *Suffolk, UK* | 017-000-121 | N/A | N/A |
| Human | Jackson ImmunoResearch  *Suffolk, UK* | 009-000-121 | N/A | N/A |
| **Primary** |  |  |  |  |
| Mouse monoclonal anti-hIL-21 | NN | NNCD0114-0000-0082-1B | 24F30A2 | IgG1 |
| Mouse monoclonal anti-hIL-21R | ZymoGenetics Inc. (ZGEN)  *Seattle, USA*  E2500 re-produced at NN  *Måløv, DK* | E2500  NNCD 0114-0000-0032-1B | 249.28.2.1.2.2  249.28.2.1.2.2 | IgG1  IgG1 |
| Rabbit monoclonal anti-hCD3 | Neomarkers | RM-9107S1 | SP7 | IgG |
| Mouse monoclonal anti-hCD20 | Dako  *Glostrup, DK* | M0755 | L26 | IgG2a |
| Mouse monoclonal anti-hCD68 | Dako  *Glostrup, DK* | M0814 | KP1 | IgG1 |
| Mouse monoclonal anti-hCD138 | Dako  *Glostrup, DK* | M7228 | MI15 | IgG1 |
| Mouse isotype specific control | R&D System  *Abingdon, UK* | MAB002 | 11711 | IgG1 |
| Mouse isotype specific control | R&D System  *Abingdon, UK* | MAB003 | 20102 | IgG2a |
| Rabbit monoclonal isotype specific control | Cell Signalling Technology  *Leiden, The Netherlands* | 3900 | DA1E | IgG |
| **Secondary** |  |  |  |  |
| Biotin conjugated Donkey anti-mouse | Jackson ImmunoResearch  *Suffolk, UK* | 715-065-150 | Polyclonal | IgG |
| Biotin conjugated Donkey anti-rabbit | Jackson ImmunoResearch  *Suffolk, UK* | 711-065-152 | Polyclonal | IgG |
| **Detection systems** |  |  |  |  |
| Peroxidase conjugated Avidin Biotin Complex (Vectastain) | Vector  *Peterborough, UK* | PK-6100 | N/A | N/A |
| Biotin conjugated Tyramide  Du Pont Blocking Reagent | Perkin Elmer  *Skovlunde, DK* | NEL700 | N/A | N/A |
| Alexa-488 conjugated Tyramide | Invitrogen  *Slangerup, DK* | T-20932 | N/A | N/A |
| Alexa-594 conjugated Tyramide | Invitrogen  *Slangerup, DK* | T-20935 | N/A | N/A |
| **Chromogenic Substrate** |  |  |  |  |
| DAB tablets | Sigma-Aldrich  *Copenhagen, DK* | D5905 | N/A | N/A |
| **Mounting Media** |  |  |  |  |
| Pertex | HistoLab  *Gothenburg, Se* | 00840 | N/A | N/A |
| FMM  Fluorescence Mounting Medium | Dako  *Glostrup, DK* | S3023 | N/A | N/A |

**Abbreviations: N/A,** not applicable. **NN,** Novo Nordisk A/S.
