# Supplementary material for: Evaluating IL-21 as a Potential Therapeutic Target in Crohn's Disease
Source: Gastroenterol Res Pract. 2018 Apr 10;2018:5962624. doi: 10.1155/2018/5962624 (PMC5914125; doi:10.1155/2018/5962624)
Supplement: Supplementary 2 — Supplementary Figure 1: exposure from AdTr colitis mice using two selected doses of mouse anti-mouse IL-21 mAb (3.3 mg/kg and 25 mg/kg) compared with simulated exposure levels. [file 5962624.f2.docx]

**Supl Figure 1 Exposure from AdTr colitis mice using two selected doses of mouse anti-mouse IL-21 mAb (3.3mg/kg and 25mg/kg) compared with simulated exposure levels**

Each dot represent an individual observation. All mice were dosed three times a week. Dark green AdTr mice (25mg/kg), dark purple AdTr mice (3.3mg/kg), light purple NMRI mice (3.3mg/kg), light green NMRI mice (25mg/kg), blue line simulated exposure 3.3mg/kg, red line simulated exposure 25mg/kg.
