# Supplementary material for: Evaluating IL-21 as a Potential Therapeutic Target in Crohn's Disease
Source: Gastroenterol Res Pract. 2018 Apr 10;2018:5962624. doi: 10.1155/2018/5962624 (PMC5914125; doi:10.1155/2018/5962624)
Supplement: Supplementary 3 — Supplementary Figure 2: transcriptional regulation in colonic biopsies after treatment with anti-IL-21 mAb. [file 5962624.f3.docx]

**Supl Figure 2 Transcriptional regulation in colonic biopsies after treatment with anti-IL-21 mAb**

Colon specimens were subjected to qPCR analysis. Open circles anti-IL-21 mAb treatment, closed squares mIgG1 treatment.
