# Supplementary material for: Evaluating IL-21 as a Potential Therapeutic Target in Crohn's Disease
Source: Gastroenterol Res Pract. 2018 Apr 10;2018:5962624. doi: 10.1155/2018/5962624 (PMC5914125; doi:10.1155/2018/5962624)
Supplement: Supplementary 4 — Supplementary Figure 3: ablation of IL-21 signalling has no effect on acute and relapsing DSS-induced colitis. Open circles indicate IL-21R−/− mice, black squares indicate IL-21R+/+ mice, and grey triangles indicate IL-21R−/+ mice. [file 5962624.f4.docx]

**Relapsing DSS model**

**Acute DSS model**

**A**

**B**

**C**

**D**

**E**

**F**

**Supl Figure 3 Ablation of IL-21 signalling has no effect on acute and relapsing DSS induced colitis**

Open circles indicate IL-21R^-/-^ mice, black squares indicate IL-21R^+/+^ mice, grey triangles indicate IL-21R^-/+^ mice. Left column acute DSS model, right column relapsing DSS model. Delta body weight change over time (days) (A-B). Disease activity index score over time (days). DAI score includes (weight change, faecal blood score and faecal stool consistency) (C-D) Colon weight length ration (E-F).
