# Supplementary material for: Evaluating IL-21 as a Potential Therapeutic Target in Crohn's Disease
Source: Gastroenterol Res Pract. 2018 Apr 10;2018:5962624. doi: 10.1155/2018/5962624 (PMC5914125; doi:10.1155/2018/5962624)
Supplement: Supplementary 5 — Supplementary Figure 4: ablation of IL-21 signalling has no significant effect on chronic DSS-induced colitis. −/− mice; black squares indicate IL-21R+/+ mice. Colon weight : length ratio (A). Endoscopic score encompassing (thickening of the colon, changes of vascular pattern, visible fibrin, granularity of mucosal surface, stool consistency) on day 20 (B). Histopathology score (C). [file 5962624.f5.docx]

**Chronic DSS model**

**A**

**B**

**C**

**Supl Figure 4 Ablation of IL-21 signalling has no significant effect on chronic DSS induced colitis**

Open circles indicate IL-21R^-/-^ mice, black squares indicate IL-21R^+/+^ mice. Colon weight length ration (A) Endoscopic score encompassing (thickening of the colon, changes of vascular pattern, visible fibrin, granularity of mucosal surface, stool consistency) on day 20 (B). Histopathology score (C).
