# Supplementary material for: Evaluating IL-21 as a Potential Therapeutic Target in Crohn's Disease
Source: Gastroenterol Res Pract. 2018 Apr 10;2018:5962624. doi: 10.1155/2018/5962624 (PMC5914125; doi:10.1155/2018/5962624)
Supplement: Supplementary 6 — Supplementary Material and Methods. [file 5962624.f6.docx]

***In-situ* hybridization on human tissue**

In brief, sections were fixed in 4% paraformaldehyde, acetylated in triethanolamine, dehydrated in increasing concentrations of ethanol and air-dried. Hybridization was performed with the ^35^S labelled probe in 10xSALTS (3 M NaCl, 0,1 M Tris, 0,1 M NaPO4, 50 mM EDTA, 0.2% Ficoll 400, 0.2% polyvinylpyrolidone (PVP 40, 40000 MW), 0.2% BSA Fraction V), 50% deionised formamide, 50% dextran sulphate, 10 ng/ml tRNA and 10 mM DTT, overnight at 47°C. Post-hybridization washes were performed in 1xSALTS, 50% formamide and 10 mM DTT for 1 hour at 62°C, 1 hour at 67°C and finally in 1xSSC with 10 mM DTT at 42°C. RNAseA (20µg/ml) post-treatment was performed for 30 min at 42°C. Sections were then washed in 0.1 SSC with 1 mM DTT for 30 min at room temperature, dehydrated through increasing concentrations of ethanol’s and air dried. Autoradiography was performed by dipping the slides in K.5 emulsion (in gel form) and exposure took place for 6 weeks in the dark at 4°C. The slides were developed in D19 developer with 1% acetic acid, 1 % glycerol and 30% Na-thiosulphate. The sections were finally counterstained with haematoxylin/eosin, dehydrated and mounted in Pertex.

**Induction of adoptive transfer colitis using CD4^+^CD25^-^ T cells**

In brief, a CD4^+^CD25^-^ cell subset was prepared from BALB/c splenocytes by positive selection of CD4^+^ T cells using Dynabeads and DETACHaBEAD (Dynal, Oslo, Norway) followed by a negative selection of CD4^+^CD25^+^ cells using the CD25 MicroBead kit (Miltenyi Biotech, Bergisch Gladbach, Germany). The purity of the cells was evaluated by flow cytometry analysis (FACS) before reconstitution. Immunodeficient SCID recipient mice were reconstituted by injection of 300,000 CD4^+^CD25^-^ T cells intraperitoneal. Peripheral blood from the mice was analysed by FACS 3 or 4 weeks after transfer and only mice with >1% CD4^+^ T cells among CD45^+^ leukocytes were included. In the prophylactic study, AdTr mice were treated with neutralizing mouse anti-mouse IL-21 mAb (25mg/kg) or isotype mouse IgG1 (25mg/kg) i.p. three times a week, from day -2 until termination of the experiment day 40. In the interventive study, AdTr mice were treated with neutralizing mouse anti-mouse IL-21 mAb (25mg/kg) or isotype mouse IgG1 (25mg/kg) i.p. three times a week, from day 21 until termination of the experiment day 52. Both antibodies were produced and purified at Novo Nordisk.

**Induction of adoptive transfer colitis using CD4^+^RB^high^ T cells**

The spleen from IL21R^-/-^C57BL/6 or IL21R^+/+^C57BL/6 littermates (8-12 weeks) was removed and splenocytes were negatively selected for CD4^+^ T cells using a cocktail of biotin-conjugated antibodies against CD8a, CD11b, CD11c, CD19, CD45R (B220), CD49b (DX5), CD105, Anti-MHC-class II, and Ter-119. The isolation of T cells was achieved by depletion of magnetically labelled cells using autoMACS columns (CD4^+^ T cell isolation kit, Miltenyi Biotec, Cat.No.130-095-248). The cell population enriched for CD4^+^ T cells were labelled with fluorochrome-conjugated antibodies and naïve CD4^+^CD25^-^CD45RB^High^ cells were sorted (>95% purity) using the BD FACSAria^TM^ II Cell Sorter (2-tube, purity, 70um nozzle, 50 psi). The recipient mice (B6-Rag2^-/-^B6.129S6-Rag2tm1Fwa N12, 9-10 weeks) were reconstituted with 400.000 cells by i.p*.* injection. Peripheral blood from all mice was subject to flow cytometric analysis 4 weeks after transfer, and only mice with CD4^+^ T cells (indicating successful transplantation of cells) were included in the study.

## Induction of Piroxicam accelerated colitis (PAC) in IL-10^-/-^ mice

IL-10^-/-^ mice had unrestricted access to piroxicam (Sigma Aldrich, Broendby, Denmark) 200 ppm homogenized in 1324 Altromin diet (Altromin, Lage, Germany) from day 0 until day 14 of the experiment, and then switched to normal Altromin 1324 chow. PAC mice were treated with neutralizing mouse anti-IL-21 mAb (25mg/kg) or isotype mIgG1 (25mg/kg) i.p. three times a week, from day -2 until termination day 17/18 of the experiment.

## Induction of DSS colitis

DSS colitis was induced in BALB/c mice by 4% DSS (ICN Biomedicals, OH) dissolved in deionised water, administrated to the mice *ad libitum* in water bottles, from day 0 until day 7 of the experiment (acute model). In the 12-day models setup (remission), mice received deionised water without DSS from day 5 until day 12. In the 21-day model setup (chronic), mice also receive a second cycle of DSS from day 12-17, and then deionised water without DSS from day 17 until day 21. DSS induced colitis mice were sacrificed at day 7, 12 or 21 of the experiment

**Flow cytometry of blood samples from mice**

50μl blood from individual mice was diluted in 50μl PBS (without Ca and Mg) containing 0.5 μg FC-Block (CD16/CD32/CD64) (2.4G2) (BD pharmingen, US). Samples were incubated at RT for 10 min and the stained with a master mix containing FITC-conjugated anti-CD4 (L3T4), PE conjugated anti-CD25 (PC61.5), PerCP-Cy5 conjugated anti-TCRbeta (H57-597), (eBioscience, Ca, US). Pacific blue conjugated anti-CD45.2

(104)(Biolegend) was also included in the master mix to identify leucocytes. All samples were stained for 30 min at 4°C. Subsequently, red blood cells were lysed and leucocytes fixed using the BD FACS lysing solution according to the manufactures instructions (BD pharmingen, US). The percentage of lymphocytes subsets was evaluated by flow cytometry using an LSRII flow cytometer (BD, US) and FACS DIVA software.

**Exposure and pharmacokinetics in mice**

A single dose pharmacokinetic study in healthy NMRI mice was completed and used for simulation of expected concentration levels following repeated dosing using a 1-compartment model (WinNonlin (Pharsight Corporation)). Two groups in a prophylactic pilot CD4^+^CD25^-^ T cell study were included for pharmacokinetic analysis. Exposure samples were collected from all mice at day 25. All exposure results were plotted against the simulated exposure. Exposure levels were estimated in the serum samples using an ELISA assay that has been setup to quantify mouse-anti-mouse-IL-21 in mouse serum. It is a sandwich ELISA that captures anti-mIL-21 between mouse IL-21 coating antigen (NN, Måløv, DK) and a HRP conjugated detection antibody against mouse IgG (P0447, Dako, Glostrup, DK). Samples were assayed in duplicates in 100 to 2,000,000 fold dilutions against an anti-mIL-21 (ZymoGenetics Inc., Seattle, USA) calibration curve. Results are reported as mean of two duplicates and CVs were below 25%. The lower limit of quantification was estimated to 65 ng/ml.

**Histological analysis on mouse tissue**

Tissue for histology was fixed in 4% paraformaldehyde (VWR – Bie & Berntsen, Herlev, Denmark) for approximately 24 hours at 4°C. Subsequently, the samples were transferred to 70% ethanol and stored at 4°C until processed for histopathology. Paraffin embedded tissue blocks were sectioned at a nominal thickness of 3 μm, and mounted on Superfrost^®^ Plus microscope slides. Subsequently, the slides were stained with haematoxylin (Amplicon, Skovlunde, Denmark) and eosin (Sigma-Aldrich, Broendby, Denmark) (H&E) for light-microscopic examination, using an Olympus AX70 microscope. The IHC staining’s were performed essentially as described for the human tissues with the following modifications: For CD3 IHC analyses slides were incubated with monoclonal rabbit anti-human CD3 antibody (RM-9107-S; SP7 Thermo Scientific), followed by amplification with the HRP-conjugated goat anti-rabbit polymer complex EnVision (Dako K4003). For neutrophil IHC staining’s staining using the rat anti-mouse antibody S100A8 (MRP-8) (My Biosource) antigen retrieval was achieved by citrate buffer pH 6.0, in a microwave oven for 15 min, followed by incubation with the primary antibody, donkey anti-rat (Jackson ImmunoResearch) and an amplification step performed by incubation with Vectastain ABC peroxidase kit according to manufacture. The chromogenic reaction was achieved with diaminobenzidin. Nuclei were counterstained with haematoxylin and the sections were rehydrated, cleared in xylene and mounted with Pertex. Control immunostaining were performed by isotype specific control, isotype at IgG2b (MCA1125 Serotec).

**qPCR of colon samples**

Colon samples were immediately placed in RNA later (Qiagen, Hilden, Germany). The samples were homogenized in 400 mL TriZol using an Ultra Turrax homogenizer (IKA, Staufen, Germany). The water phase, containing the RNA, was further purified as described in the RNeasy Kit MinElute cleanup kit manual (Qiagen). RNA qualities were confirmed on a 2100 Bioanalyzer (Agilent Technologies, Santa Clara, CA) using the RNA 6000 Nano Labchip kit (Agilent Technologies). RNA samples were reverse transcribed using the TaqMan Reverse Transcription Kit (Applied Biosystems, Foster City, CA). cDNA samples were diluted 1:10 and prepared for qPCR using TaqMan Universal PCR MasterMix (Applied Biosystems) and TaqMan Assays (Applied Biosystems/ThermoFisher Scientific). The specific assays used were; Gata3: Mm00484683_m1, Tbx21: Mm00450960_m1, Rorc: Mm01261022_m1, FoxP3: Mm00475165_m1, and 18S: Hs99999901_s1. Thermal cycling was performed on the ABI 7900HT sequence detection system.
